# Moss-like Hierarchical Architecture Self-Assembled by Ultrathin Na_2_Ti_3_O_7_ Nanotubes: Synthesis, Electrical Conductivity, and Electrochemical Performance in Sodium-Ion Batteries

**DOI:** 10.3390/nano12111905

**Published:** 2022-06-02

**Authors:** Denis P. Opra, Anton I. Neumoin, Sergey L. Sinebryukhov, Anatoly B. Podgorbunsky, Valery G. Kuryavyi, Vitaly Yu. Mayorov, Alexander Yu. Ustinov, Sergey V. Gnedenkov

**Affiliations:** Institute of Chemistry, Far Eastern Branch of the Russian Academy of Sciences, 690022 Vladivostok, Russia; dp.opra@ich.dvo.ru (D.P.O.); sls@ich.dvo.ru (S.L.S.); pab@ich.dvo.ru (A.B.P.); kvg@ich.dvo.ru (V.G.K.); 024205@inbox.ru (V.Y.M.); all_vl@mail.ru (A.Y.U.); svg21@hotmail.com (S.V.G.)

**Keywords:** hierarchical materials, nanotubes, mesoporosity, sodium trititanate, hydrothermal synthesis, conductivity, sodium-ion batteries

## Abstract

Nanocrystalline layer-structured monoclinic Na_2_Ti_3_O_7_ is currently under consideration for usage in solid state electrolyte applications or electrochemical devices, including sodium-ion batteries, fuel cells, and sensors. Herein, a facile one-pot hydrothermal synthetic procedure is developed to prepare self-assembled moss-like hierarchical porous structure constructed by ultrathin Na_2_Ti_3_O_7_ nanotubes with an outer diameter of 6–9 nm, a wall thickness of 2–3 nm, and a length of several hundred nanometers. The phase and chemical transformations, optoelectronic, conductive, and electrochemical properties of as-prepared hierarchically-organized Na_2_Ti_3_O_7_ nanotubes have been studied. It is established that the obtained substance possesses an electrical conductivity of 3.34 × 10^−4^ S/cm at room temperature allowing faster motion of charge carriers. Besides, the unique hierarchical Na_2_Ti_3_O_7_ architecture exhibits promising cycling and rate performance as an anode material for sodium-ion batteries. In particular, after 50 charge/discharge cycles at the current loads of 50, 150, 350, and 800 mA/g, the reversible capacities of about 145, 120, 100, and 80 mA∙h/g, respectively, were achieved. Upon prolonged cycling at 350 mA/g, the capacity of approximately 95 mA∙h/g at the 200th cycle was observed with a Coulombic efficiency of almost 100% showing the retention as high as 95.0% initial storage. At last, it is found that residual water in the un-annealed nanotubular Na_2_Ti_3_O_7_ affects its electrochemical properties.

## 1. Introduction

Recently, functional materials with a hierarchical architecture constructed by unique assemblies of constituent elements have gained increased attention. If such organized multilevel architectures are formed spontaneously, the process is called “self-assembling”. The character of structural integration is determined by difference in the nature of interaction forces due to which the formation of levels occurs. [1,2]. The topological benefits of hierarchical materials can provide unusual functionalities making them valuable for practical applications.

Titanium compounds are applied in a wide range of applications including paints, catalysts, plastics, coatings, sensors, hygienic products, cosmetics, pharmaceuticals, and even foods [3,4,5,6,7,8]. Yearly world titanium demand (in TiO_2_-units) is estimated around 6 million tons growing by about 3% per year [9,10,11]. Furthermore, titanium compounds are already applied in battery production (e.g., spinel Li_4_Ti_5_O_12_ that is valuable anode material for lithium storage due to its advantageous properties in long-term cycling stability) [12,13,14,15]. Looking forward to years to come, the manufacturers focus today on the development advanced energy-storage strategies based on cheaper and abundant chemicals, such as sodium-ion batteries (SIBs). One of the key challenges, hampering the SIBs commercialization, is an absence of electrode materials capable to provide required electrochemical performance but maintaining, at the same time, economic competitiveness of this technology [16,17]. Recently, transition metal oxides [18], sulfides [19], fluorides [20], phosphates [21,22], selenides [23], and nitrides [24] have been investigated as electrode materials for SIBs. Among the titanium-containing compounds, Na_2_Ti_6_O_13_, NaTi_2_(PO_4_)_3_, NaTiO_2_, TiO_2_, Na_2_Ti_4_O_9_, ATiOPO_4_ (A = NH_4_, K, Na), and Na_2_Ti_3_O_7_ turned out to be the most promising for usage as active materials in SIBs electrodes [25,26,27,28]. The benefits of Na_2_Ti_3_O_7_ include high ionic conductivity, proper sodium storage capacity (177 mA∙h/g) and suitable potential (0.2–0.3 V; can be applied as the anode material), moderate volume changes (~6%; it is less than for carbonaceous anode in LIBs (9–12%)) upon sodiation/desodiation [29,30].

This work aims to develop a facile one-pot method for preparing Na_2_Ti_3_O_7_ with a hierarchical architecture using a hydrothermal technology that meets the requirements of scalability as well as to study the features of morphology and texture, conductive, optical, electronic, and electrochemical properties of that as-prepared materials.

## 2. Materials and Methods

All reagents for experiments in this work had an analytical grade and used without further purification. Nanotube-constructed mesoporous Na_2_Ti_3_O_7_ microparticles similar to moss were prepared by a hydrothermal method through the treatment of TiO_2_ P25 (99.7%, Sigma-Aldrich, St. Louis, MO, USA) in a highly alkaline medium. In a typical procedure, 0.4 g titanium dioxide was dispersed in a 20 mL aqueous solution of sodium hydroxide (10 M) under continuous stirring. The obtained suspension was transferred into a 25 mL Teflon-lined stainless-steel autoclave. The autoclave was placed in an oven and maintained at 130 °C for 36 h. After cooling to room temperature, the resulting white suspension was precipitated by centrifugation and then many times washed with deionized water until the pH became neutral. Finally, the product was dried at 120 °C in air for 12 h (un-annealed sample denoted as NTO-120). To determine the effect of temperature on physicochemical properties, the resulting product was next annealed at 250 °C (NTO-250), 350 °C (NTO-350), and 500 °C (NTO-500) in air for 3 h.

X-ray diffraction (XRD) was carried out using a Stadi P diffractometer (STOE, Darmstadt, Germany) with Cu*K*_α_ radiation (λ = 1.5418 Å) in the 2*θ* range of 5–60° (step of 0.024°). Identification of phases was performed in EVA version 2.0 software (Bruker, Billerica, MA, USA). The morphological characteristics of materials were studied by scanning (SEM) and scanning transmission (STEM) electron microscopy on Sigma 300 VP (Carl Zeiss, Oberkochen, Germany) and S5500 (Hitachi, Tokyo, Japan) microscopes. The analysis and processing of SEM images were performed in ImageJ program developed at the National Institutes of Health (Bethesda, MD, USA) by Wayne Rasband. The elemental composition and distributions of elements were studied by energy dispersive X-ray spectroscopy (EDX) on a Carl Zeiss Merlin microscope (Carl Zeiss, Oberkochen, Germany) equipped with EDX analyzer. Nitrogen adsorption–desorption tests were performed on the Quantachrome Autosorb iQ instrument (Anton Paar, Graz, Austria). The specific surface area, pore size distribution, and pore volume were estimated using a Brunauer-Emmett-Teller (BET) and Barrett-Joyner-Halenda (BJH) models. The chemical composition was studied by X-ray photoelectron spectroscopy (XPS) on a SPECS spectrometer (SPECS, Berlin, Germany) equipped with a hemispherical Phoibos-150 analyzer using Mg*K*_α_ radiation (λ = 9.7349 Å). The binding energy scale was calibrated using the position of C 1 s peak at 285 eV. An ultraviolet-visible (UV-vis) UV2700Plus spectrophotometer (Shimadzu, Kyoto, Japan) was applied to study the electronic band structure. The experiments were carried out in the wavelength range from 200 to 800 nm. Barium sulfate was used as a reflection (white) standard. The electrophysical characteristics were investigated using a Solartron complex (Solartron Mobrey, Farnborough, Hampshire, UK) composed of SI 1260 impedance/gain-phase analyzer and 1296 dielectric interface. The materials were pressed into pellets with a diameter of 10 mm and a thickness of 0.4–0.5 mm. To provide electrical contact, pellets were coated with a conductive Dotite D-550 Silver paste. Measurements were carried out in the frequency range from 1 Hz to 20 MHz with an amplitude of 0.5 V. The bulk resistance was calculated from the high-frequency region of impedance spectra using the equivalent electrical circuit (EEC) approach. Fitting was performed using a parallel *RC*-circuit.

The electrochemical characteristics of products were determined using two-electrode ECC-STD cells (EL-Cell, Hamburg, Germany). The slurry of working electrode was prepared by mixing 90 wt.% active material and 10 wt.% Tuball^TM^ Bat NMP additive (OCSiAl, Leudelange, Luxemburg) in an appropriate amount of *N*-methylpyrrolidone. For rate capability experiments, the proportion of conductive component was adjusted to 20 wt.% by adding Super P acetylene black (Alfa Aesar, Ward Hill, MA, USA). The resulting slurry was spread in a uniform 50 µm thick layer onto an aluminium foil current collector using an AFA-III coater (MTI, Richmond, CA, USA). The foil was pre-treated in a dilute hydrochloric acid solution. The obtained electrode sheet was dried at 60 °C to a constant weight. Then, electrode disks with a diameter of 15 mm were cut out from the sheet using a GN-CP20 device (Gelon, Linyi, China). The electrochemical cells were assembled in an argon-filled glove box. Metallic sodium was used as the counter and reference electrodes. 1 M solution of NaClO_4_ in propylene carbonate with the addition of fluoroethylene carbonate (5 vol.%) was applied as an electrolyte. A separator was a GF/C glass fiber from the Whattman (Little Chalfont, Buckinghamshire, UK). The half-cells were tested on a Solartron Analytical 1400 (Solartron Mobrey, Farnborough, Hampshire, UK) and Elins P-20X8 (Elins, Zelenograd, Russia). The measurements were conducted at room temperature in the potential range of 0.01–2.5 V (vs. Na/Na^+^). Galvanostatic charge/discharge cycling was carried out at current densities of 50, 150, and 350 mA/g. During discussions, despite applying half-cells, the charge designates a sodiation process, while the discharge implies desodiation. Cyclic voltammetry (CV) curves were recorded at a potential sweep rate of 50 μV/s. Electrochemical impedance spectroscopy (EIS) spectra were recorded in the frequency range of 10^6^–10^−2^ Hz with an ac amplitude of 10 mV at the potential of 0.01 V (i.e., at a fully sodiated state). The EIS-spectra were processed with Zview software from Scribner Associates (Southern Pines, NC, USA).

## 3. Results

### 3.1. Structure, Morphology, and Conductive Properties of Moss-like Hierarchical Structure Self-Assembled by Na_2_Ti_3_O_7_ Nanotubes

**Figure 1:**
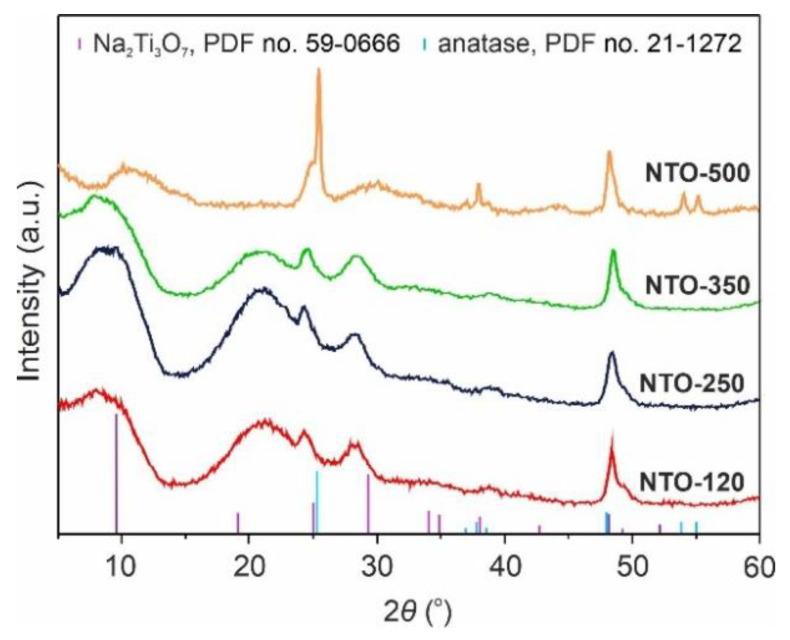
XRD patterns of Na_2_Ti_3_O_7_-based products prepared at different temperatures (120–500 °C) NTO-120, NTO-250, NTO-350, and NTO-500 products.

To study the effect of annealing temperature on the phase composition of prepared Na_2_Ti_3_O_7_ products, XRD studies were carried out. The XRD patterns for samples obtained at temperatures of 120, 250, 350, and 500 °C are illustrated in Figure 1. As it can be seen, the observed peaks in diffraction patterns are broadened presuming the nanocrystalline materials. The data show that the dominant crystal phase in NTO-120, NTO-250, and NTO-350 samples was sodium trititanate (PDF No. 59-0666) crystallizes in a monoclinic space group *P*2_1_/*m* with the parameters: *a* = 9.3987 Å, *b* = 3.7566 Å, *c* = 11.0272 Å, *β* = 101.6°. Besides, anatase titanium dioxide (PDF No. 21-1272) was identified in the analyzed samples as phase impurity. Up to annealing at 350 °C, the XRD patterns (including position, geometry, and broadening of diffraction peaks) are the same, indicating an absence of distinguished changes. On the other hand, if the calcination at the temperature of 500 °C was carried out, it would result in the irreversible phase transformation of Na_2_Ti_3_O_7_ to anatase, signed by strengthening of corresponding peaks. The obtained results are in agreement with the data of other researchers [31,32].

**Figure 2:**
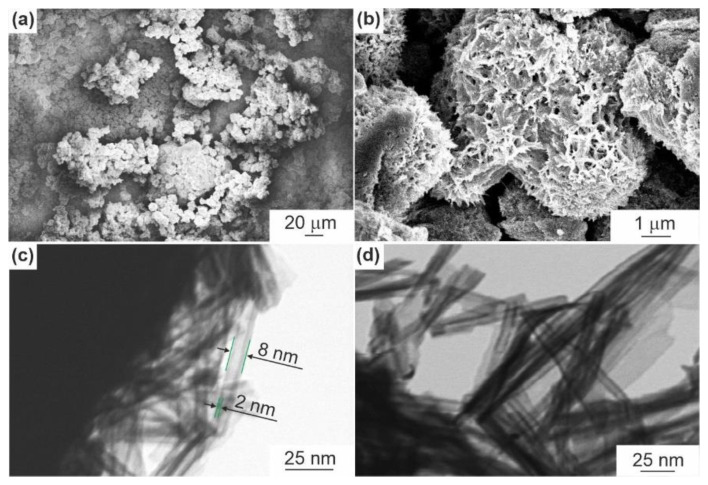
SEM-images show the microscale topography for NTO-120 sample at the magnifications of 300× (**a**) and 10,000× (**b**). STEM-imaging at the magnification of 700,000× reveals the structural hierarchy of NTO-120 (**c**) and NTO-350 (**d**) products.

For the investigation into the microstructure of synthesized materials, SEM and STEM experiments were carried out. The results reveal that samples looked identically and have a self-assembled hierarchical architecture comprising two levels of organization. As an example, in Figure 2 and Figure S1 the morphology of NTO-120 and NTO-350 products is illustrated. It is found (SEM data; Figure 2a,b and Figure S1) that first level of hierarchy is formed by microparticles of 1–10 μm in diameter possessing a rough surface. The particles look similar to moss-like lichens, called “reindeer mosses” (Figure S2).

An in-depth study (STEM mode; Figure 2c), shows that these microscale moss-like objects consist of smaller size one-dimensional hollow cylindrical nanostructures (i.e., nanotubes). The nanotubes are extremely uniform in size that is might be an important factor for usage as electrode-active materials for batteries. The outer diameter of these tubes varies in the range of 6–9 nm, the wall thickness does not exceed 2–3 nm (ultrathin). The length of nanotubes is equal to several hundred nanometers. The sample annealed at 350 °C (Figure 2d) did not demonstrate noticeable changes in microstructure, and no agglomerates of nanotubes were observed.

**Figure 3:**
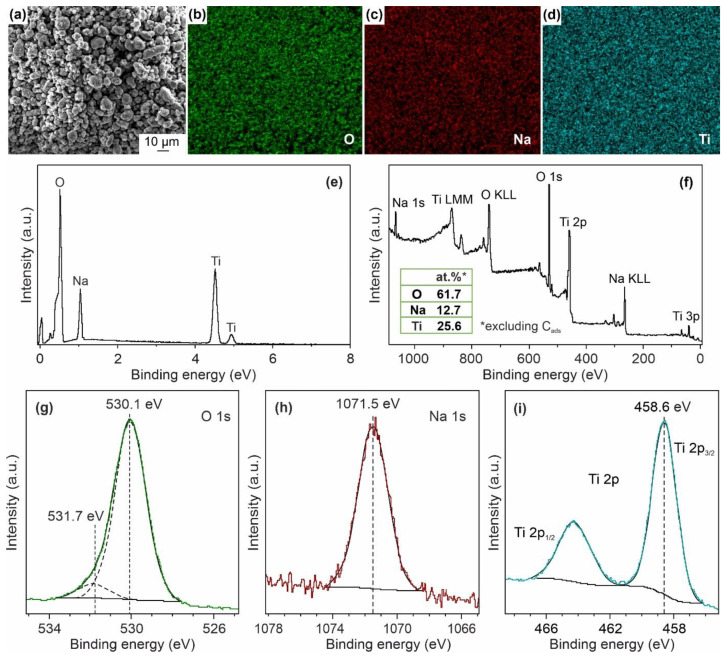
SEM-image of the analyzed area (**a**), corresponding elemental maps (**b**–**d**), and EDX-spectrum (**e**) for NTO-120 sample. XPS-spectra for the NTO-350 surface: survey scan (**f**), O 1s (**g**), Na 1s (**h**), Ti 2p (**i**). The inset in (**f**) shows the content of elements estimated from XPS peak areas.

The EDX studies give an insight on elements in the samples. It is shown that synthesized materials contain O, Na, Ti, and C in trace amounts, as displayed in Figure 3a–e using NTO-120 as an example. The maps reveal a uniform distribution of oxygen, sodium, and titanium at the products surface, as well as carbon (Figure S3), the origin of which will be clarified later using XPS.

To determine the chemical state of elements in the prepared materials, XPS experiments were performed. Figure 3 illustrated the XPS data obtained for the NTO-350 sample. The survey spectrum (Figure 3f) shows the presence of signals from Na, Ti, and O elements. The existence of carbon in XPS analyses is explained by the nanoscale morphology sensitive to environment contaminations [33,34]. In the photoelectron spectrum of O 1s (Figure 3g) two prominent peaks are detected at 530.1 eV (89%) and 531.7 eV (11%), which, respectively, associating with the oxygen bonded to metal and the oxygen of hydroxyl groups or carbonates $\left({\mathrm{CO}}_{3}^{2-}\right)$ existed at the surface. The binding energy of Na 1s (Figure 3h) is 1071.5 eV. The Ti 2p core-level XPS spectrum (Figure 3i) shows spin-orbit doublet of 2p_3/2_ (458.6 eV) and 2p_1/2_ (464.2 eV) with a separation of 5.7 eV corresponding to the +4 oxidation state of titanium. The registered positions of peaks are in agreement with that from literature for Na_2_Ti_3_O_7_ [35]. The elemental composition of NTO-350 determined from XPS peak areas (Figure 3f, inset) corresponds to the Na_1.63_Ti_3.28_O_7.07_ formula (ignoring the TiO_2_ impurity), which is close to sodium trititanate.

**Figure 4:**
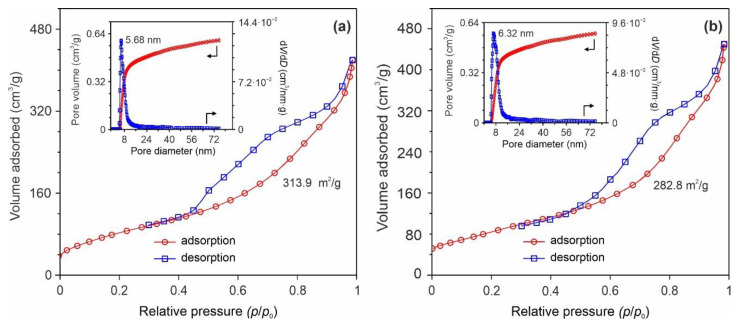
Data of adsorption measurements for NTO-120 (**a**) and NTO-350 (**b**) samples.

To obtain information about texture of synthesized Na_2_Ti_3_O_7_ materials, a low-temperature N_2_ adsorption-desorption method was used. Figure 4 represents the experimental isotherms, pore volume data, and pore size distribution curves for NTO-120 and NTO-350 samples. It can be seen that products exhibited a type IV isotherms (in terms of the IUPAC classification) with the H3 hysteresis loops starting at *p*/*p*_0_ of 0.43, which corresponds to the mesoporous materials. The pore size distribution profiles (insets in Figure 4) have narrow peaks at 5.68 nm (NTO-120) and 6.32 nm (NTO-350) further evidence that the mesoscopic scale porosity is the dominant in products. The total pore volume for samples can be estimated as 0.54–0.6 cm^3^/g. The BET specific surface area changes from 313.9 to 282.8 m^2^/g and the BJH pore volume varies between 0.54 and 0.60 cm^3^/g during treatment at temperatures in the range of 120–350 °C. Thus, it can be concluded that the obtained materials are characterized by a large specific surface area and high pore volume with a pore network formed predominantly by mesoporous channels. Interestingly, the NTO-350 sample possesses some higher pore volume as opposed to the un-annealed product. This is likely caused by the opening of pores due to dehydration (Figure S4).

Table S1 summarizing the textural characteristics and preparing techniques for different nanostructured sodium trititanate demonstrates that the synthetic procedure in this work might be useful to design and fabricate functional Na_2_Ti_3_O_7_-based materials for various fields.

**Figure 5:**
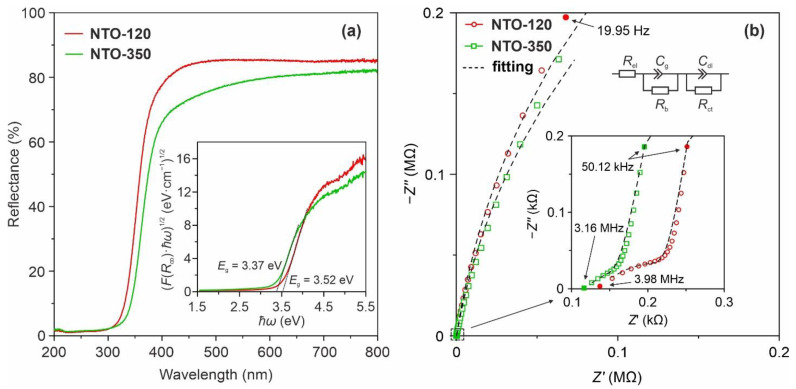
UV-vis diffuse reflection spectra (**a**) with the corresponding Tauc plots (inset) for band gap determination and impedance spectra (**b**) with the EEC used for fitting of NTO-120 and NTO-350 materials (to guide the reader’s eye high-frequency regions of spectra are shown enlarged at the right).

Figure 5a represents the UV-vis diffuse reflectance spectra of analyzed Na_2_Ti_3_O_7_ materials. According to data, the samples absorb UV-rays and have high reflectance in the visible region (λ > 400 nm), demonstrating a typical behavior for Na_2_Ti_3_O_7_, which is a wide-bandgap semiconductor. The optical band gap (*E*_g_) of the products was estimated by Tauc relation (Equation (1)) for allowed indirect transitions (γ=2) using a Kubelka–Munk model ($\alpha \sim F(R_{\infty }))$:

(1) $${\left(\alpha \cdot \hbar \omega \right)}^{1/\gamma }=A\left(\hbar \omega -E_{g}\right)$$

where α is the absorption coefficient, ℏω is the photon’s energy, A is the constant called the band tailing parameter, $F(R_{\infty })={\left(1-R_{\infty }\right)}^{2}/2R_{\infty }$ is the Kubelka–Munk function, and $R_{\infty }$ is the reflectivity.

In this way, the ${\left(F(R_{\infty })\cdot \hbar \omega \right)}^{1/2}$ against ℏω graph is plotted and then a linear section of the curve is extrapolated to the *x*-axis (an intersection point gives the *E*_g_). The Tauc plots of tested samples are shown in the inset of Figure 5a. According to estimations, the band gap of the NTO-120 sample is equal to 3.52 eV agreeing with the results of theoretical calculations (3.28 eV) [36,37] and experimental measurements (3.40–3.51 eV) [37,38,39,40] reported in the literature. Note that some authors had measured a larger band gap for Na_2_Ti_3_O_7_ (3.7–3.85 eV) [41,42,43]. After annealing at 350 °C, the band gap decreases to 3.37 eV. Probably, this may be due to change in phase ratio resulting in the construction of the Na_2_Ti_3_O_7_/anatase heterojunction structure. Further experiments are required to study this question that goes beyond the scopes of the study. On the other hand, there are many experimental data and theoretical models indicating an increase in the band gap of semiconductor nanomaterials due to decreasing their particle size [44]. This is explained by the fact that in the nanoscale state in semiconductors, the quantum confinement effect of electrons and holes occurs, which leads to an energy difference between the filled states and the empty states. Therefore, as the particle size changes, the band gap either increases or decreases.

Figure 5b illustrates the EIS spectra of hierarchical nanotube-constructed Na_2_Ti_3_O_7_ materials prepared at temperatures of 120 °C (un-annealed) and 350 °C as well as an equivalent circuit (inset) used for fitting. As it is shown the impedance profiles comprise a depressed semicircle in high frequency range and a straight line at low frequency. From the plots is observed that due to annealing the impedance tends to decrease up to 156.5 Ω (NTO-350 sample). Taking into account sample geometry, the following values of resistivity were calculated by EES fitting: 3.5 and 3.0 kΩ·cm for the NTO-120 and NTO-350, respectively. The estimated conductivity of NTO-120 product is 2.88 × 10^−4^ S/cm. Annealing at 350 °C increases the conductivity up to 3.34 × 10^−4^ S/cm. The results are consistent with the UV-Vis data. The literature analysis indicates that the observed values are rather high. Indeed, as it was found in [45], the microparticulate Na_2_Ti_3_O_7_ synthesized through the solid-state method has a conductivity of 1.19ꞏ10^−7^ S/cm (after doping with ytterbium it is increased to 1.89ꞏ10^−7^ S/cm). Table S2 presents the data calculated by modeling the experimental impedance spectra for studied materials.

### 3.2. Electrochemical Performance in Sodium Batteries of Self-Assembled Moss-like Hierarchical Architecture Constructed by Na_2_Ti_3_O_7_ Nanotubes

**Figure 6:**
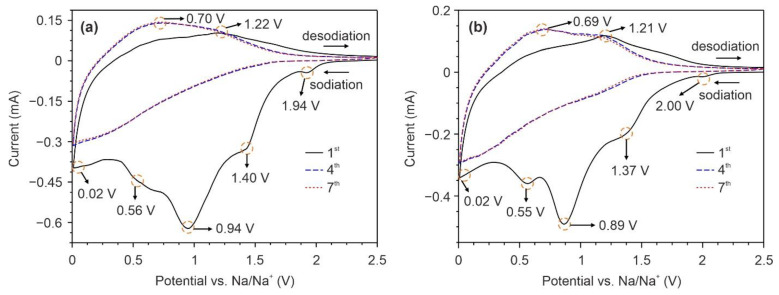
CV curves of NTO-120 (**a**) and NTO-350 (**b**) electrodes registered at a scan rate of 50 mV/s.

Figure 6 shows CV curves of the NTO-120 and NTO-350 electrodes for the 1st, 4th, and 7th cycles. In general, no noticeable differences can be found between the graphs, which is explained by the identical electrochemical reaction mechanism. During the first cycle, a series of pronounced peaks near 1.93–2, 1.37–1.4, 0.86–0.94, and 0.54–0.56 V are detected in the cathodic sweep resulting from processes that occur at the electrode surface: the accumulation (adsorption) of sodium ionic species due to Faradic redox reactions (pseudocapacitive-like behavior) [46,47] and the decomposition of electrolyte with the formation of solid electrolyte interphase layer (SEI) [48]. The cathodic peak at 0.01–0.02 V corresponds to the insertion of Na^+^ ions into the Na_2_Ti_3_O_7_ crystal lattice accompanied by a formation of Na_2+*x*_Ti_3_O_7_ and, meantime, reduction of a part of host Ti^4+^ ions to Ti^3+^ due to the charge compensation [46]. For the subsequent cycles, the contribution of irreversible processes reduces significantly, as shown in the cathodic scans of 4th and 7th CV curves. In the initial anode process, broad peaks near 0.7 and 1.2 V are observed in CVs of NTO-120 and NTO-350 samples. Upon cycling, the anodic region evolves: the peak located at 0.7 V becomes more intense (it is stabilized after the 4th cycle), whereas the peak at 1.2 V, on the contrary, decreases. According to the previous works [49,50], the region of lower potentials refers to the extraction of sodium ions from the Na_2_Ti_3_O_7_ lattice with the oxidation of trivalent Ti to tetravalent. The decrease in intensity of peak around 1.2 V during cycling likely results from incompletely reversible adsorption of Na^+^ ions in the pores of the materials and/or inside the nanotubes [46,47].

**Figure 7:**
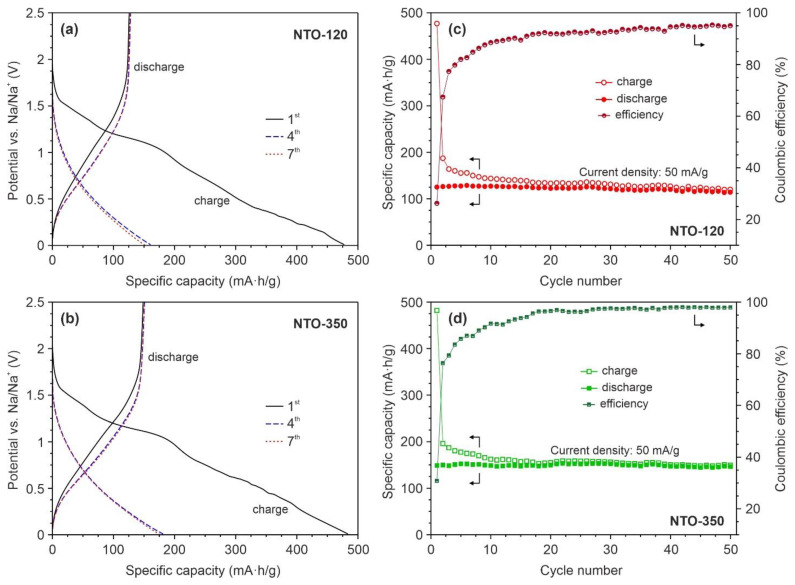
Charge-discharge voltage profiles of the 1st, 4th, and 7th initial cycles (**a**,**b**) and cycleability (**c**,**d**) for the NTO-120 and NTO-350 electrodes at a current density of 50 mA/g.

**Figure 8:**
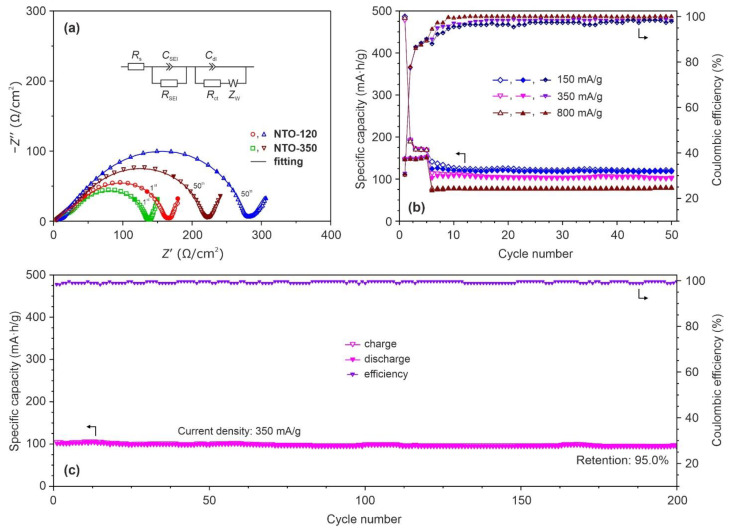
Impedance spectra registered at a fully sodiated state (at a potential of 0.01 V) during 1st and 50th cycles for analyzed materials (filled symbols denote the frequencies of 10^3^, 10^2^, and 10^−2^ Hz) with an electrical equivalent circuit (inset) applied for fitting (**a**), rate capability data (charge and discharge capacities are marked by filled and open symbols, Coulombic efficiencies are designated by semi-open symbols) after five initial formation cycles at 50 mA/g (**b**), and prolonged cycling performance at 350 mA/g (**c**) for the NTO-350 electrode.

The results of galvanostatic charge/discharge tests for NTO-120 and NTO-350 electrodes at a current density of 50 mA/g (~0.3 *C*) are shown in Figure 7. As can be seen, the cycling data are consistent with that of CV studies. Indeed, the initial charge curves of both samples (Figure 7a,b) exhibit a number of inflections at the potentials close to positions of peaks in the first cathodic CV scans. The initial charge capacities of NTO-120 and NTO-350 are comparable and reach about 480 mA∙h/g. During the first discharge process, the NTO-350 electrode delivers a specific capacity of 149 mA∙h/g, higher than NTO-120 (125 mA∙h/g). Analogously to CV measurements, noticeable irreversible losses are observed for samples mainly due to the electrolyte reduction and SEI film formation [51]. Next, the initial charge/discharge profiles differ from that of subsequent cycles. On the contrary, the curves of the 4th and the 7th cycles are almost coincident, implying the same electrochemical reaction mechanisms (Equation (2)). The reversible (discharge) specific capacities of analyzed materials after the 7th cycle are stabilized at approximately 127 mA∙h/g (NTO-120) and 151 mA∙h/g (NTO-350), which corresponds to deintercalation of about 1.4 and 1.7 sodium ions per formula unit of sodium titanate (*x* in Equation (2)). The corresponding Coulombic efficiencies are equals to 84.8% and 86.9%. As it will be shown below (Figure 8a), such moderate efficiency in initial cycles is associated with the evolution of SEI layer.

Na_2_Ti_3_O_7_ + *x*Na^+^ + *x*e ↔ Na_2+*x*_Ti_3_O_7_ (2)

where *x* is the amount of Na^+^ incorporated into Na_2_Ti_3_O_7_ matrix (can reach from 2 to 3.5 [35,52,53]).

Figure 7c,d illustrates the evolution in specific capacities of tested materials during 50 charge/discharge cycles. The data show that upon cycling a slowdown in the rate of capacity degradation is observed for both electrodes. At the same time, it is clear that NTO-120 sample possesses a worse electrochemical performance. The drop in capacity of such electrode from the 7th to 50th cycle was about 10%, while for NTO-350 that did not exceed 3%. Observed differences in the behavior of NTO-120 and NTO-350 can probably be explained by unequal degree of dehydration. Indeed, according to TGA data (Figure S4), the total weight loss for NTO-120 and NTO-350 in the range from room temperature to 1000 °C is about 15.6 and 8.5%, respectively. Besides the water exists in various states in the analyzed products, namely absorbed molecules (the portion is the same for NTO-120 and NTO-350), free water, and H_2_O molecules located in the interstitial cavities (only in un-annealed material). The literature overview reveals that free and coordinated water effects differently on the electrochemical performance in SIBs [54,55,56,57,58]. After the 50-fold cycling, the NTO-120 and NTO-350 maintained, respectively, around 114 and 146 mA∙h/g. The corresponding Coulombic efficiencies increase to above 95.0 and 97.9%.

Figure 8a depicts the EIS-spectra of NTO-120 and NTO-350 samples registered at a fully sodiated state (0.01 V) during 1st and 50th cycles.

The impedance spectra show depressed semicircles in the high-to-middle frequency range, followed by a low-frequency sloping line. It can be seen from graphs that upon cycling the radii of semicircles increase for both samples. At the same time, a difference in semicircles sizes is observed obviously from one to another studied materials. Moreover, it rose during cycling, as illustrated in EIS-plots. In order to analyze the observed features, next, the fitting of impedance curves was performed using the equivalent electric circuit (Figure 8a, inset) that involves the following components: *R*_s_ is the ohmic resistance of the cell, *R*_SEI_ and *C*_SEI_ are the resistance and geometric capacitance of a SEI film, *R*_ct_ and *C*_dl_ are the charge transfer resistance and double layer capacitance at an electrode/electrolyte interface, *Z*_W_ is the diffusion resistance. Note that bring of all of these elements into EES is dictated by the physical context within analyzed cells and supported by the goodness of fitting (χ^2^ ≤ 10^−4^). Table S3 represents the evolution of parameters for NTO-120 and NTO-350 based cells during cycling. The results show that in an initial period (first cycle) the NTO-350 possesses some lower values of *R*_SEI_ and *R*_ct_ (33.7 and 98.2 Ω/cm^2^, respectively) as compared to that of NTO-120 (51.4 and 108.0 Ω/cm^2^). After the 50th cycle, the difference in the resistance of a SEI layer as well as in the charge transfer resistance between samples increases (NTO-350: *R*_SEI_ = 65.5 Ω/cm^2^, *R*_ct_ = 151.8 Ω/cm^2^; NTO-120: *R*_SEI_ = 110.7 Ω/cm^2^, *R*_ct_ = 167.2 Ω/cm^2^). The obtained data correlate with that of CV-experiments (Figure 6) and charge/discharge tests (Figure 7). The same results without explanation were reported previously in [59] for the nanotubular Na_2_Ti_3_O_7_ prepared at 130 and 150 °C. As seems, the observed features in electrochemical performance of such materials can be explained by different amounts of water. It is interesting to mention that before cycling (Figure S5), a relatively high impedance (exceeding those on the 1st and 50th cycles) of NTO-120 and NTO-350 samples is measured. Similar data were presented earlier in [42,59]. The observed phenomenon is explained by the fact that at the first cycle end most of the material’s surface is covered with a SEI film reduces its surface energy and, hence, decreases the interfacial impedance (as supposed in [44]).

Rate capability experiments (Figure 8b) show that the performance of NTO-350 sample is stable under cycling with increased current densities. Indeed, it exhibits a reversible capacity of ~118 mA∙h/g (a capacity drops of about 19% as opposed to that at 50 mA/g) with a Coulombic efficiency of 97.9% at a cycling rate of 150 mA/g (~0.85 *C*). When the current load rises to 350 mA/g (~2 *C*), a discharge capacity drop of approximately 14% (to ~102 mA∙h/g at the 50th cycle) as well as a Coulombic efficiency growth to 98.3% were observed for NTO-350 electrode. Hence, the rate of capacity degradation from cycle to cycle slows down when an applied current density is increased. Similar behavior of Na_2_Ti_3_O_7_ during cycling at higher rates is described in [50,51] and attributed to variations in SEI thickness or composition. When the NTO-350 was measured by 50 cycles of charge/discharge at a current density of 800 mA/g (~4.5 *C*), it exhibited approximately 79 mA∙h/g with an almost 100% Coulombic efficiency.

Figure 8c displays the prolonged cycling performance of the NTO-350 electrode (continuation of the tests shown in Figure 8b) at a current load of 350 mA/g. Under continuous Na^+^ ions insertion/extraction during 200 cycles the analyzed material keeps a considerable capacity of about 97 mA∙h/g with the retention as high as 95.0% initial storage indicating its superior cyclic and adequate rate performances for SIBs.

## 4. Conclusions

In this work, we have developed an efficient strategy for the preparation of moss-like Na_2_Ti_3_O_7_ frameworks assembled from ultrathin one-dimensional nanotubes with an outer diameter of 6–9 nm, a wall thickness of 2–3 nm, and a length of several hundred nanometers. The material has a unique structure similar to moss at the micro level and a high specific surface area (314 m^2^/g). The product is characterized by a porous texture (0.54 cm^3^/g) with a narrow pore size distribution near 5.7 nm. It was also found that the material is thermally stable up to 350 °C: its textural characteristics almost do not change (nanotube agglomeration was not observed), the specific area and pore volume after heat treatment remain at the level of 283 m^2^/g and 0.60 cm^3^/g, respectively, pores with a diameter of 6.3 nm are predominated. It is found that the phase transformation of Na_2_Ti_3_O_7_ into anatase occurs near 500 °C. The moss-like nanoarchitecture of sodium trititanate improves electrical conductivity reaching 2.88ꞏ10^−4^ S/cm. Next, as it was found, heat treatment at 350 °C affects the electrical properties of Na_2_Ti_3_O_7_: the conductivity increases three-fold to 3.34ꞏ10^−4^ S/cm. The SIBs-type devices based on a hierarchically-organized nanotubular Na_2_Ti_3_O_7_ possess promising cyclic and rate characteristics. A capacity of more than 95 mA∙h/g after the 200th cycle is preserved even at a current density of 350 mA/g with a Coulombic efficiency of almost 100%. It was found that the degree of dehydration determines the electrochemical performance of the nanotubular Na_2_Ti_3_O_7_. As we believed the reported strategy of hierarchically-organized Na_2_Ti_3_O_7_ nanotubes may have a great potential for other fields (e.g., biomedicine, photocatalysis, and ion exchanger applications).

## Data Availability

The data presented in this study are available on request from the corresponding author.

## Supplementary Materials

The following supporting information can be downloaded at: https://www.mdpi.com/article/10.3390/nano12111905/s1, Figure S1: SEM-images at the magnifications of 378× (a) and 17,180× (b) for the NTO-350 sample; Figure S2: Close-up view for a white reindeer moss lichen from the Rondvassbu National Park (Norway, Europe). The photo was taken by Martin Schneiter and is available for license from the iStock website; Figure S3: Elemental mapping of carbon for the NTO-120 material; Figure S4: Thermogravimetric analysis curves of the NTO-120 and NTO-350 samples; Figure S5: Nyquist plots of NTO-120 and NTO-350 electrodes before cycling (i.e., so-called «fresh» cells); Table S1: Method of synthesis and textural characteristics of some promising functional materials based on Na_2_Ti_3_O_7_ [60,61,62,63,64,65]; Table S2: Calculated EIS parameters of NTO-120 and NTO-350 samples; Table S3: The EIS-spectra fitting results of NTO-120 and NTO-350 electrodes.
