# Supplementary material for: Moss-like Hierarchical Architecture Self-Assembled by Ultrathin Na2Ti3O7 Nanotubes: Synthesis, Electrical Conductivity, and Electrochemical Performance in Sodium-Ion Batteries
Source: Nanomaterials (Basel). 2022 Jun 2;12(11):1905. doi: 10.3390/nano12111905 (PMC9182444; doi:10.3390/nano12111905)
Supplement: Supplementary file 1 [file nanomaterials-12-01905-s001.zip › nanomaterials-1716111-supplementary.pdf]

Moss-like hierarchical architecture self-assembled by ultrathin $Na_2Ti_3O_7$ nanotubes: Synthesis, electrical conductivity, and electrochemical performance in sodium-ion batteries

Denis P. Opra, Anton I. Neumoin *, Sergey L. Sinebryukhov, Anatoly B. Podgorbunsky, Valery G. Kuryavyi, Vitaly Yu. Mayorov, Alexander Yu. Ustinov and Sergey V. Gnedenkov

Institute of Chemistry, Far Eastern Branch of the Russian Academy of Sciences, 690022 Vladivostok, Russia; dp.opra@ich.dvo.ru (D.P.O.); sls@ich.dvo.ru (S.L.S.); pab@ich.dvo.ru (A.B.P.); kvg@ich.dvo.ru (V.G.K.); 024205@inbox.ru (V.Y.M.); all_vl@mail.ru (A.Y.U.); svg21@hotmail.com (S.V.G.)

* Correspondence: anton_neumoin@ich.dvo.ru; Tel.: +7-(924)-338-8153

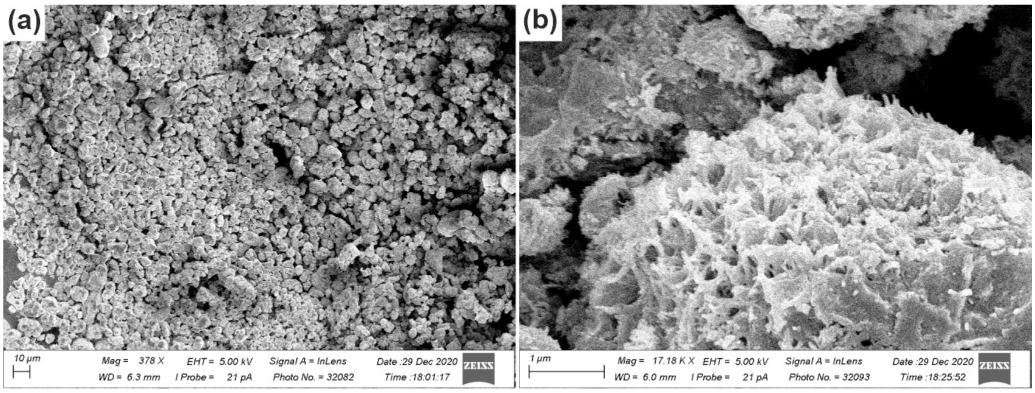

Figure S1. SEM-images at the magnifications of 378× (a) and 17,180× (b) for the NTO-350 sample

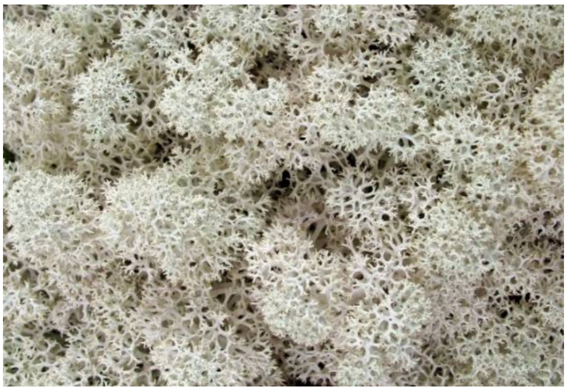

Figure S2. Close-up view for a white reindeer moss lichen from the Rondvassbu National Park (Norway, Europe). The photo was taken by Martin Schneiter and is available for license from the iStock website

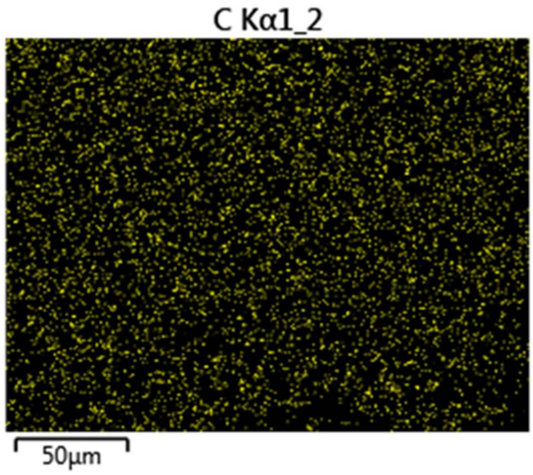

Figure S3. Elemental mapping of carbon for the NTO-120 material

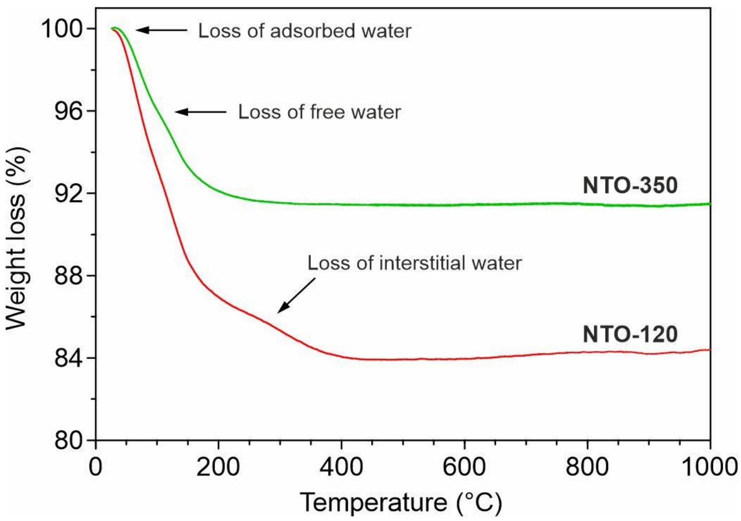

Figure S4. Thermogravimetric analysis curves of the NTO-120 and NTO-350 samples

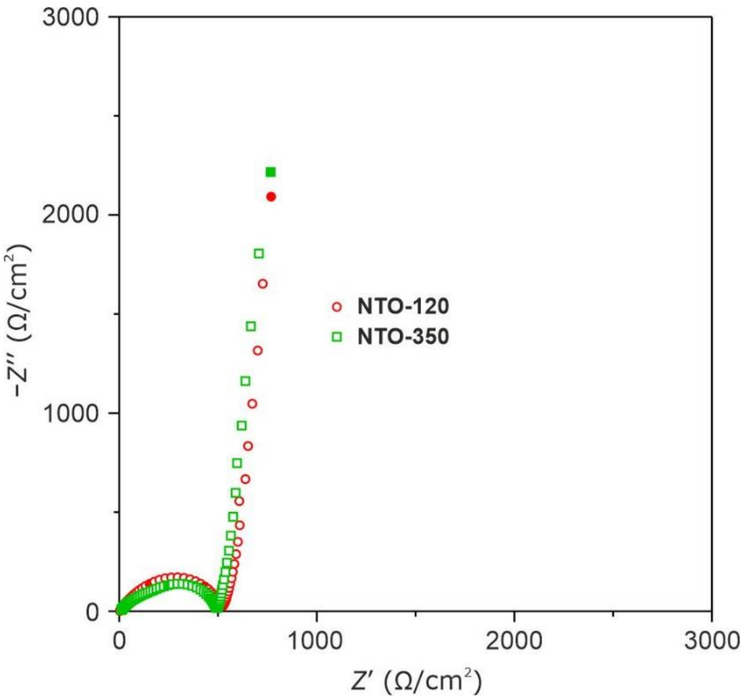

Figure S5. Nyquist plots of NTO-120 and NTO-350 electrodes before cycling (i.e., so-called «fresh» cells). Closed symbols denote the frequencies of 10^3, 10^2, and 10^{-2} Hz

Table S1. Method of synthesis and textural characteristics of some promising functional materials based on $Na_2Ti_3O_7$

$Na_2Ti_3O_7$-based structure	Method of preparing	Specific surface area, m^2/g	Pore volume, cm^3/g	Reference and year
$Na_2Ti_3O_7$ nanowires embedded into 3D graphene networks	Two-step solvothermal	61.9	0.33 (mesoporous)	[60] 2016
Self-doped $Na_2Ti_3O_7$ microparticles coated with carbon	Sol-gel followed by thermal treatment in H_2/Ar	134.3	–	[61] 2018
$Na_2Ti_3O_7$ nanofibers encapsulated in carbon	Hydrothermal with annealing in presence of MXene ($Ti_3C_2T_x$)	132.9	(mesoporous)	[62] 2019
Mesoporous $Na_2Ti_3O_7$ microspheres with rigid framework	Hydrothermal	62.9	0.4	[63] 2019
Heterostructure based on $Na_2Ti_3O_7$ nanotubes and V_2O_5 nanoparticles	Two-step hydrothermal	283.7	0.32 (mesoporous)	[64] 2018
$Na_2Ti_3O_7$ nanosheets	Supercritical hydrothermal	69.5	–	[65] 2016
Coral-like hierarchical nanotube-assembled $Na_2Ti_3O_7$	Hydrothermal	282.8	0.60 (mesoporous)	this work

Table S2. Calculated EIS parameters of NTO-120 and NTO-350 samples

Sample	C_g		R_b (Ω)	C_{dl}		R_{ct} (Ω)	χ^2	WSS
	Q_g (S/cm·s^n)	n		Q_{dl} (S/cm·s^n)	n			
NTO-120	$2.89{\cdot}10^{-8}$	0.64	225.6±1.2	$5.41{\cdot}10^{-8}$	0.91	$0.94{\cdot}10^{6}$	0.000291	0.027085
NTO-350	$4.02{\cdot}10^{-6}$	0.36	156.5±1.5	$6.84{\cdot}10^{-8}$	0.89	$0.80{\cdot}10^{6}$	0.000224	0.021276

Table S3. The EIS-spectra fitting results of the NTO-120 and NTO-350 electrodes

Sample		After 1st charge (at a fully sodiated state; 0.01 V)	After 50th charge (at a fully sodiated state; 0.01 V)
NTO-120	R_s (Ω/cm^2)	4.3	4.5
	R_{SEI} (Ω/cm^2)	51.4	110.7
	R_{ct} (Ω/cm^2)	108.0	167.2
NTO-350	R_s (Ω/cm^2)	4.3	4.4
	R_{SEI} (Ω/cm^2)	33.7	65.5
	R_{ct} (Ω/cm^2)	98.2	151.8
